# Black Phosphorus Transistors with Near Band Edge Contact Schottky Barrier

**DOI:** 10.1038/srep18000

**Published:** 2015-12-15

**Authors:** Zhi-Peng Ling, Soumya Sakar, Sinu Mathew, Jun-Tao Zhu, K. Gopinadhan, T. Venkatesan, Kah-Wee Ang

**Affiliations:** 1 Silicon Nano Device Laboratory, Department of Electrical and Computer Engineering, National University of Singapore, 4 Engineering Drive 3, 117583 Singapore; 2 Centre for Advanced 2D Materials and Graphene Research Centre, National University of Singapore, 6 Science Drive 2, 117546 Singapore; 3 NUSNNI-NanoCore, National University of Singapore, 117576 Singapore

## Abstract

Black phosphorus (BP) is a new class of 2D material which holds promise for next generation transistor applications owing to its intrinsically superior carrier mobility properties. Among other issues, achieving good ohmic contacts with low source-drain parasitic resistance in BP field-effect transistors (FET) remains a challenge. For the first time, we report a new contact technology that employs the use of high work function nickel (Ni) and thermal anneal to produce a metal alloy that effectively reduces the contact Schottky barrier height (Φ_B_) in a BP FET. When annealed at 300 °C, the Ni electrode was found to react with the underlying BP crystal and resulted in the formation of nickel-phosphide (Ni_2_P) alloy. This serves to de-pin the metal Fermi level close to the valence band edge and realizes a record low hole Φ_B_ of merely ~12 meV. The Φ_B_ at the valence band has also been shown to be thickness-dependent, wherein increasing BP multi-layers results in a smaller Φ_B_ due to bandgap energy shrinkage. The integration of hafnium-dioxide high-k gate dielectric additionally enables a significantly improved subthreshold swing (SS ~ 200 mV/dec), surpassing previously reported BP FETs with conventional SiO_2_ gate dielectric (SS > 1 V/dec).

Progressing towards the international technology roadmap for semiconductors^1^ demands devices with ever shrinking dimensions. This comes with a diverse set of challenges that need to be addressed. These include the search for alternative high mobility channels, achieving low contact resistivity, low parasitic resistance and capacitance, continual effective oxide thickness scaling, and the integration of low bandgap channels materials such as Ge, III-V and two-dimensional (2D) materials among others. The growing interest in 2D materials has started from the intense research of graphene^2^,^3^, in which the ability to isolate individual, atomically thin layers from the 3D form (graphite) has revealed unique electronic and optical properties from its bulk form^4^,^5^. Some unique properties of graphene include room-temperature electron mobility^6^ reaching 2.5 × 10^5^ cm^2^ V^−1^ s^−1^, a high intrinsic strength of 130 GPa^7^, high thermal conductivity^8^ above 3000 W mK^−1^, and high electrical stability^9^ among others, easily surpassing those reported on silicon-based materials. Coupling these unique advantages with a scalable deposition approach such as the roll-to-roll deposition onto flexible substrates^10^ has opened up new potential applications in the fields of transparent conductive layers, bio-applications, photonics, nanoelectronics, sensors, high-frequency transistors, and novel electronic devices such as Van der Waals heterostructures^11^.

Despite the advantages presented by graphene, the lack of a bandgap in pristine graphene limits its application in field-effect transistors (FETs) due to its low on/off current switching ratios, and thus cannot be effectively turned off. Various efforts to introduce a bandgap has been reported^12^,^13^,^14^,^15^,^16^ to varying success, at the expense of increased complexity and decreased carrier mobilities. To address the bandgap limitation, a different class of 2D materials known as transition metal dichalcogenides (TMDCs)^5^ had been reported which comprises of a transition metal element from group IV, V or VI and a chalcogen (S, Se or Te) in a “MX_2_” configuration. The metal atoms are sandwiched between two hexagonal planes of chalcogen atoms through an ionic-covalent interaction, and the materials can range from metallic to semiconducting. The intriguing aspect of semiconducting TMDCs lies in the thickness-dependent properties such as the transition from an indirect bandgap in the bulk to a direct bandgap in the monolayer form. An example is MoS_2_ in which the energy bandgap increases from an indirect gap of 1.3 eV in the bulk form to a direct bandgap of over 1.9 eV in the monolayer form. A tremendous increase in the luminescence quantum efficiency by a factor exceeding 10^4^ is experimentally confirmed as compared with the bulk material^17^. The use of the large bandgap TMDCs materials for field-effect transistors can easily outperform graphene-based FETs, with the first implementation of a monolayer MoS_2_ top-gated transistor by Kis and co-workers^18^ exhibiting excellent current on/off ratio (~10^8^), room-temperature mobility exceeding 200 cm^2^ V^−1^ s^−1^ and a subthreshold swing of 74 mV/dec.

In comparison to the graphene and TMDC class of materials, black phosphorus (BP) is another possible candidate for nanoelectronics and optoelectronics devices. Similar to the two material classes mentioned earlier, the individual layers of the black phosphorus are held together by weak van der Waals interlayer interaction, allowing easy isolation of these layers by the micromechanical exfoliation approach. Unlike the TMDCs class of materials, black phosphorus films are reported to exhibit a direct bandgap characteristics for all layer thicknesses, tunable from ~0.3 eV in the bulk form^19^,^20^ and increases with reducing layer thicknesses, approaching ~2 eV for the monolayer form^21^. Combining the thickness-dependent tunable direct bandgap characteristics with experimentally demonstrated record high field-effect hole mobility^22^ approaching 1000 cm^2^ V^−1^ s^−1^ , black phosphorus is a promising candidate for both nanoelectronics and optoelectronics applications in the future.

For these atomically thin 2D materials to be successfully integrated into high-performance devices, the major performance-limiting factors must be identified and optimized. Achieving low contact resistivity at the source and drain regions are among the key challenges in which it is highly dependent on the metal/semiconductor contact interface and is a topic of keen research. For the case of MoS_2_, both low work function and high work function metals ranging from scandium (Φ_m_ = 3.5 eV) to platinum (Φ_m_ = 5.9 eV) has been explored^18^,^23^,^24^,^25^,^26^,^27^. Schottky barrier heights as low as ~30 meV was determined through temperature-dependent studies using a suitably low work function metal such as scandium. The metal/MoS_2_ interface is further evaluated to be strongly affected by the Fermi level pinning close to the conduction band of MoS_2_, leading to the preference of lower work function metal candidates to form improved contacts. A lower Schottky barrier also enhances the effective field-effect mobility values, attributable to the lower contact resistance and higher current injection. On the other hand, various BP-based FETs has also been recently reported^22^,^28^,^29^,^30^,^31^,^32^,^33^,^34^,^35^,^36^ in both top-gated and back-gated configurations utilizing different gate dielectrics such as SiO_2_, HfO_2_ and Al_2_O_3_. Unlike MoS_2_, the metal/BP interface is not strongly affected by the Fermi level pinning effect^31^ and the choice of the contact metal can effectively tune the Schottky barrier heights at the interface. This combined with the ability to switch between p-type and n-type conduction through the control of the gate and drain bias allows ambipolar operations and the potential for CMOS integrations. As part of the CMOS integration process, a thermal annealing process is typically required to improve the contact quality, but which is seldom investigated in previously reported BP FETs till date.

In this work, we present the realization of high performance BP field-effect transistors with a back-gate configuration on a ~15 nm thick BP channel. When integrated with a high-k gate dielectric, the BP FET realized in this work achieved a high field-effect mobility of 413 cm^2^V^−1^s^−1^ and a significantly improved subthreshold swing (SS) of ~200 mV/dec, outperforming previously reported BP FETs based on SiO_2_ gate dielectric (SS > 1 V/dec). Next, a systematic study of the thermal anneal conditions on the contact Schottky barrier heights on both thick and thin BP crystal is conducted. Utilizing a temperature dependent I–V study of the BP devices, the influence of thermal anneal conditions on the hole Schottky barrier can be extracted. The contributions from the thermionic emissions and thermal-field assisted tunneling components are also analyzed. Transmission electron microscopy (TEM) and energy-dispersive X-ray spectroscopy (EDX) measurements further confirmed the formation of a low-resistivity Ni_2_P metal alloy which contributes to the near band edge hole Schottky barrier height.

## Results

**Figure 1:**
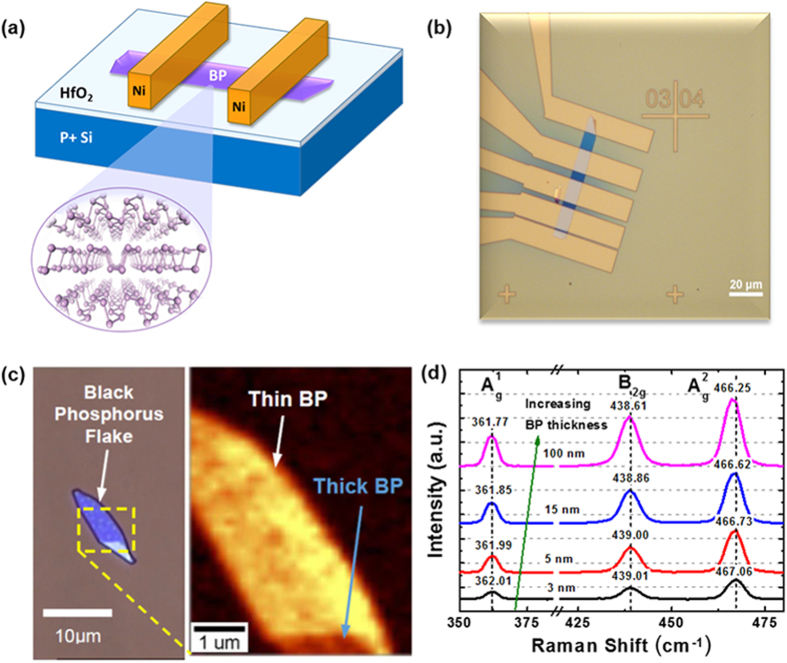
(**a**) Schematic of the BP FET utilizing a HfO_2_ high-k gate dielectric and Nickel metal electrodes in a back-gated configurations. (**b**) Optical image of the BP FET with multiple electrodes with different channel lengths which facilitates TLM measurements for the extraction of contact resistance. (**c**) A typical Raman spectra map showing the different intensity for the thin and thick BP films. (**d**) Raman spectra of the BP film showing shifts in the A^1^_g_, B_2g_, and A^2^_g_ phonon modes with increasing BP thicknesses from ~3 nm to ~100 nm, consistent with ref. [38,^39^].

Figure 1a show a schematic of the completed black phosphorus FET. The inset shows the layered BP material held together by weak van der Waals forces, with an interlayer spacing^33^ of ~5 Å. Figure 1b shows a typical top-view optical image of a completed BP FET with multiple metal electrodes with varying channel lengths from 3 μm to 21 μm. This facilitates the performance study of back-gated BP FETs with variable channel lengths, as well as the extraction of contact and channel resistance. The thickness of the BP flake in Fig. 1b is determined by atomic force microscopy (Bruker, Dimension FastScan®) to be ~15 nm. Thicker BP layers are generally preferred for device fabrication due to the improved field-effect mobility demonstrated experimentally^22^,^32^. With a thicker BP channel, the charge impurity scattering at the interface can be more effectively reduced due to the screening effect. A tradeoff is present though with increasing interlayer resistance and poorer current turn on/off characteristics with increasing number of layers.

The uniformity of a typical BP film can be deduced from the Raman measurements (WITec, Alpha300R), where BP exhibits thickness-dependent Raman intensities (Fig. 1c) and shifts in the 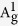, 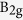 and 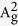 phonon modes (Fig. 1d) that are in line with previous reports^34^,^36^,^37^,^38^,^39^. In all cases, prior to measurements, the Raman instrument is calibrated to give a consistent Si Raman peak position of 520.91 ± 0.1 cm^−1^. Subsequently, temperature dependent electrical measurements (QuantumDesign, PPMS) were performed on both as-fabricated samples and after these samples were subjected to a thermal annealing process (Jipelec, Jetstar). The thermal anneal conditions were selected as 100 °C, 200 °C, and 300 °C for one minute under nitrogen (N_2_) ambient to study the influence of temperature on the contact Schottky barrier height.

**Figure 2:**
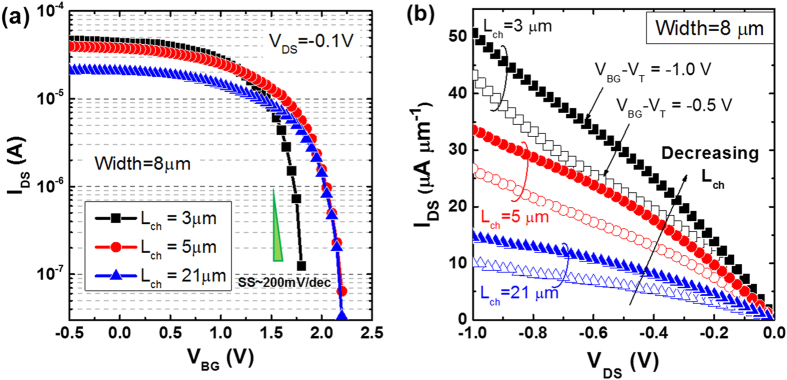
(**a**) Excellent transfer characteristics were achieved in our BP FETs with HfO_2_ high-*k* gate dielectric, showing a low subthreshold swing (SS) of ~200 mV/dec for different channel lengths (L_ch_). (**b**) Output characteristics of the same BP FETs for different channel lengths (L_ch_) and gate overdrive voltages (V_BG_-V_T_). Reducing L_ch_ and increasing V_BG_-V_T_ yield higher drive current performance.

Figure 2a shows the transfer characteristics of the BP FETs for a drain voltage V_DS_ of −100 mV and varying channel lengths from 3 μm to 21 μm. Clear p-type characteristics was observed, and low subthreshold swing of ~200 mV/dec was achieved across different channel lengths. These values outperform previously reported BP FETs fabricated on thick SiO_2_ dielectric which has subthreshold swing exceeding 1 V/dec. The use of a high-*k* gate dielectric strongly reduces the scattering from Coulombic impurities^40^, resulting in an improved on/off ratio. A current on/off ratio of ~3 orders is observed for our devices, although further improvement is expected with even thinner BP layers. The corresponding field-effect mobility was extracted as 413 cm^2^ V^−1^ s^−1^ for a channel length of 21 μm. Figure 2b shows the output characteristics of the same BP FETs for different channel lengths and gate overdrive voltages. At low drain bias, the I_DS_-V_DS_ curves are linear indicating good contact properties at the Ni/BP interface. Even after increasing the V_DS_ up to −1 V, current saturation was not observed, implying that even higher performance is achievable.

**Figure 3:**
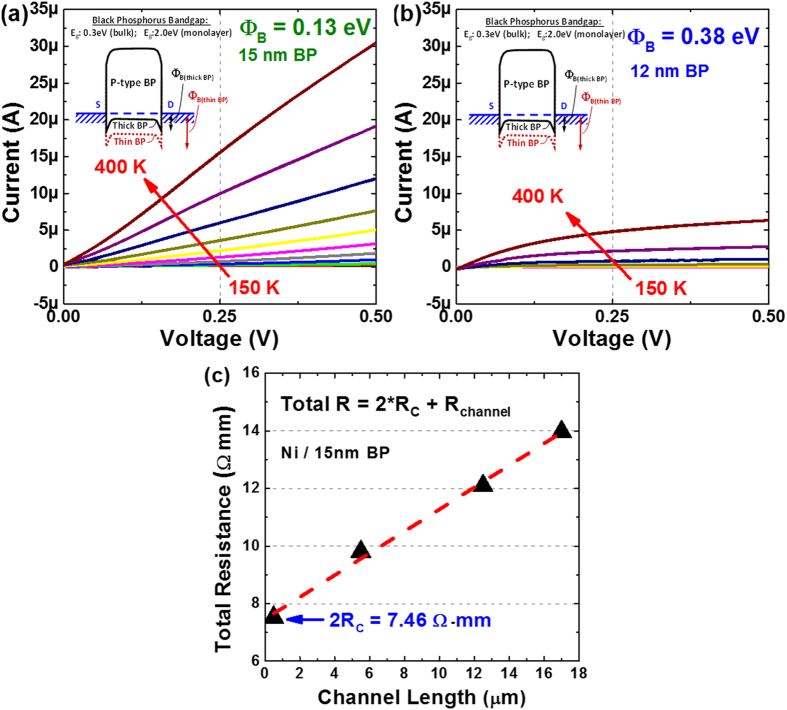
Temperature dependent I–V curves as measured from the Ni contacts on (a) thick (15 nm) and (b) thin (12 nm) BP films. A difference in the BP thickness has been shown to affect the band alignment and thus Schottky barrier heights (see inset). (**c**) Using TLM measurements, the contact resistance was extracted to be ~3.73 Ω-mm for the Nickel contacts on a 15 nm BP film.

Note worthily, it was found that the contact Schottky barrier height exhibits a dependence on the BP crystal thickness. When nickel (Ni) electrodes with a high work function of 5.1 eV were formed on BP with varying thicknesses and thus bandgap energies, the band alignment has been shown to result in a difference in the Schottky barrier heights. This is shown in the temperature dependent I-V curves as plotted in Fig. 3a, where a higher current was measured from the thick BP film (~15 nm) due to a smaller hole Φ_B_. In contrast, Fig. 3b showed that when the BP thickness was reduced to ~12 nm, a much lower current was measured which indicates an increase of the hole Φ_B_ due to an enlarged BP bandgap energy. Using the transfer length method (TLM), the Ni/BP contact resistance for the thicker BP film (15 nm) was extracted to be 3.73 Ω-mm without any gate bias (Fig. 3c). Further reduction in the contact resistance could be expected under negative back-gate bias^31^. This stems from the increased hole carriers density under the metal contact, which will lead to a narrower Schottky barrier width, hence allowing improved holes injection from Ni to the valence band of BP.

**Figure 4:**
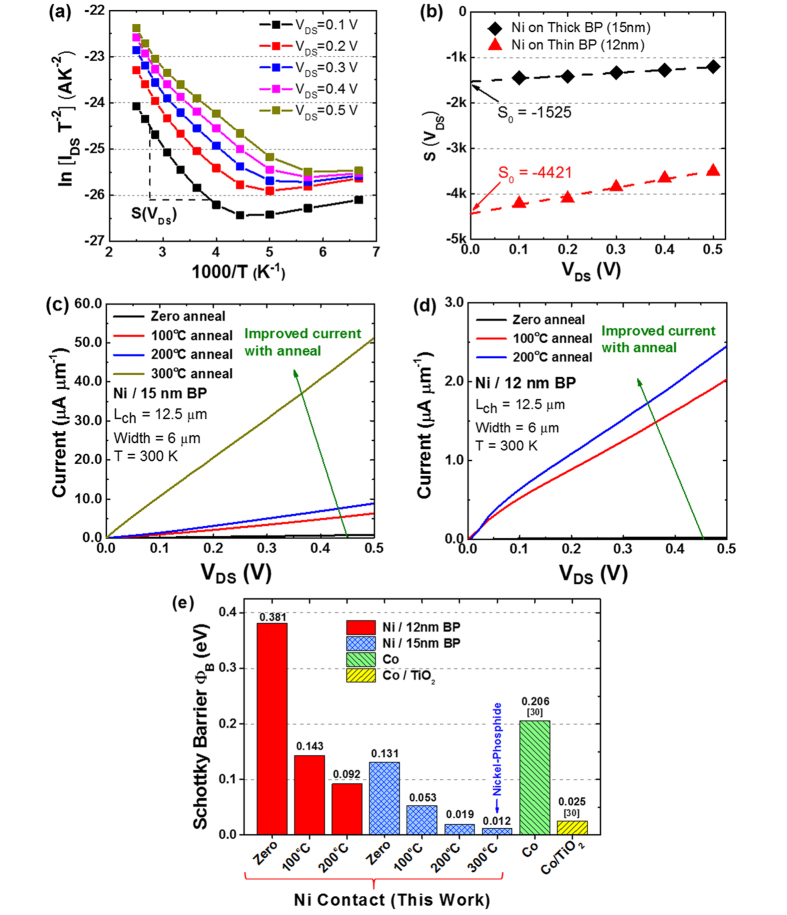
(**a**) Arrhenius plot of *ln(I*_*DS*_*/T*^*2*^) versus *1/T* for different channel bias *V*_*DS*_, and the extraction of bias dependent slope *S(V*_*DS*_) at the linear region. (**b**) The extrapolation of *S(V*_*DS*_) to zero applied channel bias give *S*_*0*_ and the Schottky barrier height at thermal equilibrium is deduced. (**c**,**d**) Improved I–V characteristics with thermal anneal for contacts formed on both thick (15 nm) and thin (12 nm) BP films. (**e**) Comparison of the extracted Schottky barrier heights to earlier reports by Kamalakar *et al*.^30^ using Co and Co/TiO_2_ tunnel contacts. Thermal anneal causes the interaction of Ni and BP crystal which produces a nickel-phosphide (Ni_2_P) alloy that de-pins the metal Fermi level towards the valence band edge. This leads to a reduction of contact Schottky barrier height to as low as 12 meV.

The Schottky barrier height at the metal/BP interface can be extracted from the temperature dependent I–V measurements and analyzed using the thermionic emission equation^41^, which can be expressed as


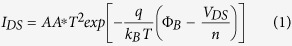


where *A* is the contact area, A* is the Richardson constant, *T* is the temperature, *q* is the electron charge, *k*_*B*_ is the Boltzmann constant, *Φ*_*B*_ is the Schottky barrier height, *V*_*DS*_ is the applied channel bias, and *n* is the ideality factor. By plotting the Arrhenius plot of *ln(I*_*DS*_*/T*^*2*^) versus *1/T* for different channel bias *V*_*DS*_, and getting the bias dependent slope *S(V*_*DS*_) at the linear region as shown in Fig. 4a, the extraction of the Schottky barrier height at thermal equilibrium can be obtained by extrapolation to zero applied channel bias as per Fig. 4b.

Figure 4c shows an improvement in the I–V characteristics for the thick BP film (15 nm) after the sequential increase in annealing temperatures from zero to 300 °C. Figure 4d shows the same trend for the thinner BP film (12 nm) after the sequential increase in annealing temperatures from zero to 200 °C. This originates from the improvement of the metal/BP contact resistance as evident from the reduced hole Schottky barrier height (Fig. 4e). This allows an increased current injection for the same applied channel bias. It is worthy to note that the Schottky barrier height of the thinner BP film (12 nm) reduces from 381 meV to 92 meV after a 200 °C anneal. In contrast, for the thicker BP film (15 nm), the Schottky barrier height was reduced from 131 meV to near valence band edge of merely 12 meV after a 300 °C anneal. These extracted values compare favorably with other reports, for example that from Kamalakar *et al*.^30^ using Co (206 meV) and Co/TiO_2_ tunnel contacts (25 meV). In these tunnel contacts, a TiO_2_ tunnel barrier is inserted between the Cobalt contact and the BP film to avoid the conductivity mismatch problem and to avoid contact-induced spin relaxations. However, there is a need to optimize the thickness of the tunnel barrier to achieve both good uniformity and low interface resistance.

## Discussion

**Figure 5:**
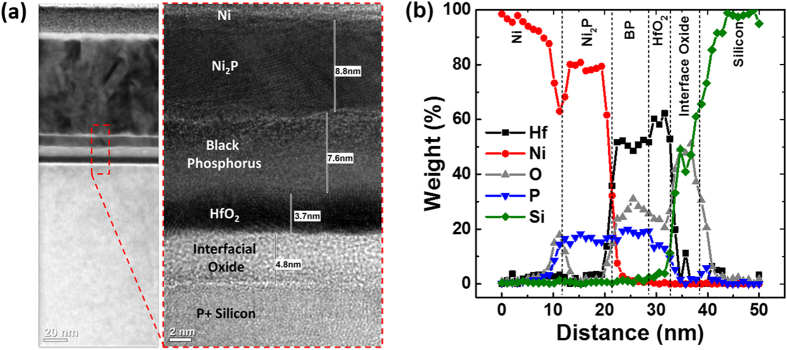
(**a**) High resolution transmission electron microscopy (HRTEM) micrographs of the BP FETs after a 300 °C thermal anneal, showing the formation of a nickel-phosphide (Ni_2_P) metal alloy with a low resistivity phase, which effectively reduces the contact Schottky barrier heights; (**b**) The energy-dispersive X-ray (EDX) spectroscopy scan confirms the formation of Ni_2_P metal alloy.

To understand the underlying mechanism for the observed near band edge hole Schottky barrier after the thermal anneal process, the devices were analyzed using transmission electron microscopy (TEM) and energy-dispersive X-ray (EDX) spectroscopy as shown in Fig. 5a,b, respectively. It was found that when Nickel electrodes were subjected to an anneal process at elevated temperature (300 °C, one minute, nitrogen ambient), the Ni would react with the underlying BP crystal to produce a nickel-phosphide (Ni_2_P) metal alloy. This is confirmed by the TEM analysis as shown in Fig. 5a, where an ultra-thin (~8.8 nm) Ni_2_P metal alloy was uniformly formed on the BP crystal. The formation of this Ni_2_P metal alloy could serve to reduce the Ni/P interface defects and the metal induced gap states, hence leading to Fermi level de-pinning towards the valence band edge which reduces the hole Schottky barrier height. This is similar to the discussion on the role of chalcogen vacancies defects in monolayer transition metal dichalcogenides on the Fermi level pinning at contacts as elaborated by Guo *et al*.^42^. By reducing the defect concentrations in the 2D materials, both the metal induced gap states and the Fermi level pinning issue are mitigated. It is worth noting that the resulting BP film thickness also reduces due to the consumption of BP film during the formation of Ni_2_P metal alloy.

The composition of the nickel-phosphide can be determined from the EDX scan as shown in Fig. 5b. From the weight percentage of the EDX spectrum, Ni and P constitute to 80% and ~20%, respectively, of the total weight. Using this information, and the fact that the atomic mass of Ni and P is 58.6934u and 30.973761u, respectively, the atomic percentage of Ni and P are calculated as 67.8% and 32% respectively. Hence, the stoichiometric ratio (Ni/P) can be determined as 2.11 which is very close to 2, or equivalently the Ni_2_P metal alloy with a low-resistivity phase. After source/drain patterning by EBL, there is a finite time interval before the next step of Ni deposition by electron-beam evaporation. Thus a small degree of exposure to moisture in the environment is unavoidable, leading to the formation of a thin NiO_x_ phase at the Ni/BP interface. With increased anneal temperature, the Ni atoms were found to diffuse through the thin NiO_x_ interface layer and interacted with the BP layers to form the Ni_2_P alloy. Although the final BP thickness is 7.6 nm, it is to be noted that the formation of the low resistivity Ni_2_P layer between the Ni and BP layer will dominate in the reduction of contact Schottky barrier due to Fermi level de-pinning effect. Additionally, the out-diffusion of the Hf and O atoms from the underlying HfO_2_ dielectric is observed, which is likely to be aggravated with higher annealing temperatures. The BP atoms have also been found to out-diffuse to the underlying HfO_2_ dielectric film at elevated temperature, as confirmed by the energy-dispersive X-ray (EDX) scan in Fig. 5(b).

**Figure 6:**
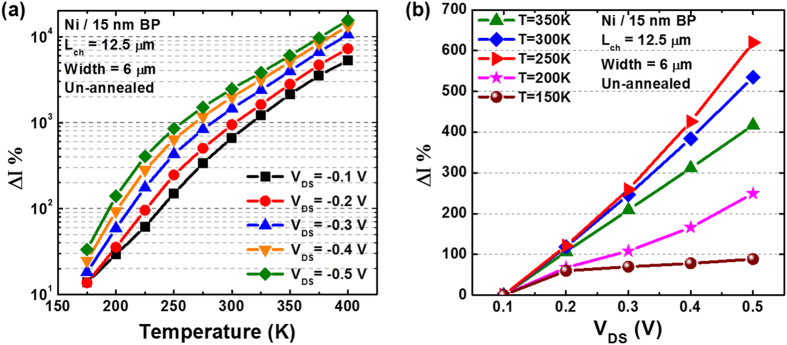
(**a**) Temperature-dependent current changes as a function of applied bias, which shows an exponential increase with increasing temperature; (**b**) Bias-dependent current changes as a function of temperature, showing a linear increase with bias for temperature exceeding 200 K.

Another key observation from Fig. 4a is that the Arrhenius plot of *ln(I*_*DS*_*/T*^*2*^) versus *1/T* saturates for lower temperatures, which suggests that the current injection is not dominated by thermal emission alone^43^. According to Appenzeller *et al*.^43^, for a simulated current injection dominated by thermal emission only, the corresponding Arrhenius plot would be a straight line, while a gradual decrease in the slope with decreasing temperatures indicates the contribution from thermally assisted tunneling as well. To gain an insight into the relative contributions from the thermionic emission and thermal-field tunneling components, Fig. 6a shows a comparison of the temperature-dependent current changes while Fig. 6b shows the bias-dependent current changes for the 15 nm thick BP sample in the as-fabricated state. In Fig. 6a, using the lowest temperature (150 K) as reference, for an applied channel bias as low as 0.1 V, the current increases exponentially with temperature and exceeds 50 times the reference value at the maximum measurement temperature of 400 K. In comparison, Fig. 6b show that using the lowest channel bias (0.1 V) as reference, as the channel bias increases, the increase in the current tends to follow a linear trend instead for temperature exceeding ~200 K, and is less than 7x times the reference value for all channel bias values and temperatures. It is clearly evident from these graphs that the contribution from the thermal-field assisted tunneling is also present amid at a much lower degree. However with additional thermal anneal, the formation of the low resistivity metal/BP alloy interface layer would serve to reduce the contact Schottky barrier further where thermionic emission is expected to dominate over the tunneling component. While the data presented here do not take into consideration the application of a back-gate bias, it is to be noted that further tuning of the metal/BP Schottky barrier is possible via the back-gate bias, allowing even higher levels of current injection with the same channel bias.

In summary, this work demonstrated a novel approach in realizing a near band edge contact Schottky barrier height in BP field-effect transistors via the use of metal alloy. The significant improvement in hole Φ_B_ is attributed to the formation of nickel-phosphide (Ni_2_P) alloy with a low-resistivity phase, which arises from the interaction between nickel and BP crystal at elevated temperatures. This results in a record low hole Φ_B_ of merely ~12 meV due to the de-pinning of metal Fermi level towards the valance band edge. The Schottky barrier height has also been shown to further reduce with increasing multi-layer BP thickness as a result of bandgap energy shrinkage. When integrated with high-k gate dielectric, an enhanced hole mobility and a significantly improved subthreshold swing were achieved over previously reported BP FETs with conventional SiO_2_ gate dielectric. Our experimental findings could pave the way for the use of BP channel in next generation transistor applications.

## Methods

### Fabrication

Using a single-sided polished planar p-doped silicon wafer with a resistivity of ~1 Ω-cm, the native oxide is removed using a 2% concentration dilute HF dip prior to the deposition of hafnium dioxide gate dielectric by the ALD process (CambridgeNanoTech, Savannah Systems). Due to finite waiting period between the pre-clean step and the ALD process, a thin interfacial layer was formed. The deposition rate of the hafnium dioxide films was estimated at ~1 Å/cycle, utilizing the tetrakisethylmethylamino hafnium (TEMAH), and water precursors at a deposition temperature of 250 °C for a total of 40 cycles. For every cycle, the TEMAH precursor and the water precursors are pulsed at 0.015 seconds and 0.01 seconds respectively, followed by a waiting time of 10 seconds. During deposition, nitrogen is utilised as the carrier gas, and is flowing at 20 sccm. This is followed by the micromechanical exfoliation of the black phosphorus crystals (from HQGraphene) using a two-step dry exfoliation method as described by Andres *et al*.^44^. This method utilises both the cleanroom tape and the PDMS stamp in sequential exfoliation and transfer. Such approach produces transferred BP flakes with significantly improved film uniformity and lower tape residues as compared to the tape transfer approach alone. This is followed by a sequential cleaning in acetone followed by isopropyl alcohol (5 mins each) before the deposition of the metal contacts (120 nm Nickel) by a combination of electron beam lithography (JEOL, JBX-6300FS) and electron-beam evaporation (Oerlikon, Univex 450B). The remaining photoresist is then removed via the lift-off process using acetone to obtain the BP FETs as sketched in Fig. 1a. To protect the BP layer from photo-oxidation in ambient conditions^39^, an additional surface passivation by a 30 nm thick SiO_2_ film is adopted over the entire BP flake right after the source/drain electrodes are deposited.

## Additional Information

**How to cite this article**: Ling, Z.-P. *et al*. Black Phosphorus Transistors with Near Band Edge Contact Schottky Barrier. *Sci. Rep*. **5**, 18000; doi: 10.1038/srep18000 (2015).
